# Cyclic Fatigue Resistance of WaveOne Gold, ProDesign R and ProDesign Logic Files in Curved Canals In Vitro 

**DOI:** 10.22037/iej.v12i4.17494

**Published:** 2017

**Authors:** Sílvio Emanuel Acioly Conrado de Menezes, Shirley Machado Batista, Juliana Ouro Preto Lira, Gabriela Queiroz de Melo Monteiro

**Affiliations:** a *Dental School, University of Pernambuco FOP-UPE, Tabatinga, Camaragibe-PE, Brazil*

**Keywords:** Cyclic Fatigue, ProDesign Logic, ProDesign R, WaveOne Gold

## Abstract

**Introduction:**

Endodontic instruments are developed to provide a better cleaning of the root canal system and reduce its risk of fracture. The aim of this study was to evaluate the instrumentation time and cyclic fatigue resistance of WaveOne Gold, ProDesign R and ProDesign Logic files.

**Methods and Materials:**

Thirty Nickel-titanium (NiTi) rotary instruments were divided into 3 groups (*n*=10). ProDesign Logic file 25/0.06 was used in continuous rotation after glide path preparation. WaveOne Gold 25/0.07 and ProDesign R 25/0.06 files were used in reciprocating motion. Every file instrumented 3 standardized artificial canals. The average time, the number of cycles (NCI) and cyclic fatigue resistance of each file were determined through the number of cycles to failure (NCF) in a stainless-steel device. The total amount of cycles to fracture was also calculated (NCI+NCF). Data was analyzed using the Kruskal-Wallis and Mann-Whitney tests.

**Results:**

The instrumentation time of the ProDesign Logic file was significantly lower when compared to the other files (*P*=0.019). The longest times to failure were presented by ProDesign Logic (182.07 sec) and ProDesign R (152.38 sec) files. The same differences were observed for the NCF (910.37 and 761.93). The WaveOne Gold group presented a lower NCF as well as a smaller sum of NCI+NCF (748.33) that was statistically significant when compared to the other groups (*P*<0.05) respectively.

**Conclusion:**

The use of continuous rotational motion in canals with a glide path in the ProDesign Logic group led to shorter instrumentation time. The cyclic fatigue resistance of ProDesign R and Logic instruments was superior to WaveOne Gold. The thermal treatment of the instrument’s alloy, its cross section and the glide path seems to influence the cyclic fatigue resistance.

## Introduction

Canals with a sharp curvature remain challenging as they present a greater degree of difficulty during instrumentation [1]. Nickel-titanium (NiTi) instruments were introduced to endodontics to shape root canals more efficiently and reduce the incidence of procedural errors [2]. However, unexpected separation of rotary instruments without apparent deformation is common occurrence among professionals [3, 4].

Innovations such as instrument design, manufacturing processes and thermo-mechanical treatment of alloys are developed to increase fatigue failure resistance as well as providing greater safety for use [5-7]. The effectiveness and lifespan of these instruments are determined, among other factors, by operational speed and torque [8]. The reciprocating motion in combination with a single-file instrumentation was suggested to make the preparation of the root canal faster due to an increased resistance to cyclic fatigue of the files and reduced chance of cross-contamination [9, 10]. 

WaveOne Gold system (Dentsply Maillefer, Ballaigues, Switzerland) uses a single-file technique with a reciprocating motion and it is manufactured through a thermal treatment process called GOLD [11, 12]. This technology, according to the manufacturer, has been suggested to improve the cyclic fatigue resistance and flexibility of the instruments. Its golden color is a result of a thermal cycling procedure used, with a repeated slow heating and cooling of the files [13]. The WaveOne Gold files are available in different tip sizes and tapers 20/0.07 (small), 25/0.07 (primary), 35/0.06 (medium) and 45/0.05 (large). These files have parallelogram cross-sectional design with 2 cutting edges and an inactive tip. The cutting edges have an 85^°^ angle, that contacts the wall of the canal at alternating points which are active when they rotate counterclockwise [13].

The reciprocating kinematics and single-file technique are also used in the ProDesign R (Easy Equipamentos Odontológicos, Belo Horizonte, MG, Brazil). According to the manufacturer, this system is available in two different tip sizes and tapers 25/0.06 and 35/0.05, with a modified S-shaped cross-sectional design, an inactive tip, variable helical angle, with two cutting blades and a counterclockwise motion similar to WaveOne Gold. Its manufacturing process is based on the CM-Wire thermal treatment. This treatment is known to produce a better arrangement of the crystalline structure resulting in improved flexibility which can be considered advantageous [14-16]. It provides instruments with little or no shape memory, making it more flexible [16], resistant to cyclic fatigue and torsional failure [14].

ProDesign Logic System (Easy Equipamentos Odontológicos, Belo Horizonte, MG, Brazil) has been designed to fit any motor in the market, as it can be used in continuous rotation. According to the manufacturer, this instrument features metallurgic characteristics similar to ProDesign R, differing only in its motion. The ProDesign Logic has cutting ability when rotated in a clockwise direction. Even when used in a continuous rotation motion, the screwing effect is reduced. The ProDesign Logic system aims to unite the concepts of a single-file and comprises of shaping files (25/0.06, 30/0.05, 35/0.05, 40/0.05) and glide path files (25/0.01, 30/0.01, 35/0.01, 40/0.01).

Several studies have evaluated the cyclic fatigue of NiTi rotating files [11, 17, 18]. However, although WaveOne Gold primary, ProDesign R and ProDesign Logic seem to have improvements for their mechanical properties, there are no studies comparing their resistance to cyclic fatigue. Thus, the present study aimed to evaluate the fracture resistance of WaveOne Gold primary, ProDesign R (25/0.06) and ProDesign Logic (25/0.06) files. The null hypotheses was that there is no difference between the systems regarding instrumentation time and cyclic fatigue resistance.

## Materials and Methods

The sample calculation was performed using BioEstat 5.3 (Instituto de Desenvolvimento Sustentável Mamirauá, Manaus, Brazil) by selecting the Independent samples *t* test. An *α* type error of 0.05, a *β* power of 0.80, and a 1:1 ratio was stipulated. The ideal sample size per group was 7 to note significant differences. A sample size of 10 was used to compensate possible outlier values that might lead to sample loss.

The characteristics of the files evaluated in this study are shown in table 1.

Before testing, all instruments were inspected under a stereomicroscope (SteREO Discovery, V12, ZEISS, Germany) under 16× magnification to observe the presence of deformities or defects. No signal was detected.

***Canal preparation***

Experimental groups were formed by each evaluated instrument (*n*=10). Each file, instrumented three acrylic resin artificial canals (IM Brazil Ltda., São Paulo, SP, Brazil). The artificial canal had a standard size, curvature and conicity with a 60^°^ angle of curvature with 5 mm radius and the center of the curvature were 5 mm from the tip of the instrument.

The working length (WL) was defined after exploration of the artificial canals with stainless steel K-files #10 (WL=17 mm). During instrumentation, the canals were irrigated with 2 mL of distilled water (Iodontosul-Industrial Odontológica do Sul Ltda., Porto Alegre, Brazil) to remove resin remains. The canals were then filled with a detergent lubricant solution based on Sodium Lauryl Sulfate (Tergipol, Biodynamic Chemistry and Pharmaceutical LTDA, Ibiporã, PR, Brazil). 

All the preparations were performed by a single operator using a 1:6 reduction handpiece (Sirona Dental Systems GmbH, Bensheim, Germany) operated by a torque-controlled motor (Silver Reciproc, VDW, Munich, Germany). For the WaveOne Gold primary and ProDesign R files, the "Reciproc All" pre-setting program was selected. For the ProDesign Logic files, the "Dr's" pre-setting program at 350 rpm and 4 N torque were used. For ProDesign Logic files, a 25/0.01 rotating file was used for glide path at 350 rpm and 1N of torque. The overload and distribution of undesirable forces in the files were controlled respecting three gently in-and-out pecking motion, with short amplitude strokes. At each cycle, the flutes were cleaned with a gauze and the canal was irrigated with distilled water and the detergent lubricant solution. Upon reaching WL, the file was cleaned and reserved for further cyclic fatigue testing.

The average instrumentation time (in sec) of each file to prepare the artificial canals was recorded by a digital timer by a single operator. The number of cycles that the instrument made to prepare the canal (NCI) was calculated according to the following formula [19]: NCI=rpm × instrumentation (sec)/60 sec.

***Cyclic fatigue test***

**Figure 1:**
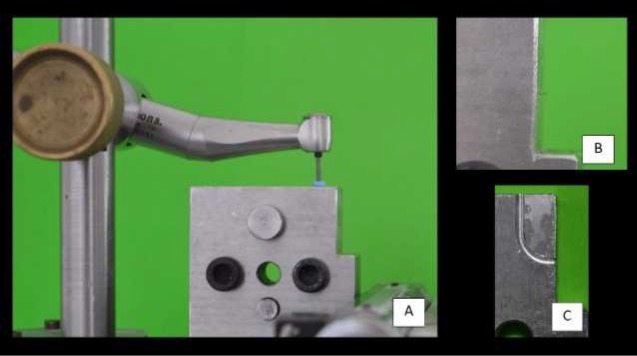
*A)* Counter-angle x file ratio x stainless steel apparatus used in the cyclic fatigue test; *B)* File tip out of the canal; *C)* Characteristics of the artificial canal geometry

The cyclic fatigue test was performed on a stainless-steel device simulating a canal of 1.5 mm diameter, 90^°^ curvature angle with 5 mm radius, and a 5 mm curvature center from the tip of the instrument [10]. A custom-made support was used to keep the steel canal, and the contra-angle static during use, allowing only free rotation of the file (Figure 1). A lubricating oil WD-40 (AP Winner Ind. Com. De Prod. Químicos LTDA, Ponta Grossa, PR, Brazil) was used to minimize the heat generated by the friction between the metal surfaces.

The time from the beginning of the instrumentation motion to the fracture of the instrument was recorded. It was possible to establish the number of cycles of the instrument to failure (NCF) using the following formula [19]: NCF= rpm × time to failure (sec)/60 sec

The total use of the file to failure was obtained by the sum of NCI and NCF.

***Statistical analysis***

Data was analyzed using SPSS program in version 13 (Statistical Package for the Social Sciences, Chicago, USA). Descriptive statistics were obtained, and non-parametric Kruskal-Wallis and Mann-Whitney tests were used for comparison between groups. The level of significance was set at 0.05.

## Results

The results for the criteria studied can be seen in table 2. The Kruskal-Wallis test did not show differences among the groups only for NCI (*P*=0.073). The instrumentation time of the ProDesign Logic file was significantly lower when compared to the other files (*P*=0.019). The time to failure revealed longer times for the ProDesign Logic and ProDesign R files with 182.07 sec and 152.38 sec, respectively. These systems also presented the highest values for the NCF (910.37 and 761.93, respectively). 

The WaveOne Gold primary group showed a smaller NCF and also a smaller sum of NCI+NCF (748.33). For both evaluated items, WaveOne Gold primary showed a statistically significant difference with the other ProDesign groups (*P*<0.05).

## Discussion

Important advances were made in the field of mechanized instrumentation leading to the insertion of numerous systems with innovative designs and differentiated metallurgy [20]. However, while WaveOne Gold primary system has been extensively evaluated [11-13], little research has been conducted on evaluating ProDesign R [21, 22] and ProDesign Logic files [23]. Several studies have used human teeth extracted to study cyclic fatigue [24-26]. However, such choice may have bias in the search results since the canals tend to vary considerably in their anatomy [27] which represents a challenge in terms of sample standardization [28]. For this reason, laboratory studies with simulated canal in resin blocks assure control of experimental conditions, ensuring only the variables of interest are analyzed.

Both hypotheses of this study were rejected. Statistically significant differences were observed between the instrumentation times and cyclic fatigue resistance of the studied files.

ProDesign Logic had shorter time preparation than the other groups. Continuous rotational motion, high torque and use of a 25/0.01 file to perform the glide path were probably determining factors for its shorter instrumentation time. Also, during the instrumentation, the fracture of two ProDesign R files and five WaveOne Gold primary files occurred. In this study, ProDesign Logic instrumented all canals without fractures. We believe that such incident directly influenced the unfavorable outcome of NCF and NCI+NCF of the WaveOne Gold primary group because the fractured files were discarded reducing the sample. These good results were already found in another previous study with the ProDesign Logic system [23].

**Table 1:** Characteristics of the tested instruments

	**Length (mm)**	**Tip (mm)**	**Taper (%)**	**Motion**	**Machining direction**	**Cross section**
**WOG**	25	0.25	0.07	Reciprocating	CCW	Parallelogram-shaped
**PDR**	25	0.25	0.06	Reciprocating	CCW	Modified S-shaped
**PDL**	25	0.25	0.06	Rotary	CW	Modified S-shaped

**Table 2:** Mean values and Standard Deviation of the Preparation time (sec), the number of cycles for each instrumentation (NCI), Time to instruments failure(sec), the number of cycles to failure (NCF) and the sum of NCI and NCF (*n*=10

	**Preparation time**	**NCI**	**Time to failure**	**NCF**	**NCI+NCF**
**PDL**	64.55 (5.04)^a^	376.59 (29.43)	182.07 (94.42)^b^	910.37 (472.10)^b^	1286.96 (488.40)^b^
**PDR**	102.65 (58.71)^b^	513.28 (293.57)	152.38 (97.95)^b^	761.93 (489.75)^b^	1275.21 (685.86)^b^
**WOG**	96.72 (25.71)^b^	483.61 (128.57)	52.95 (61.08)^a^	264.76 (305.42)^a^	748.37 (364.31)^a^

*There were statistical differences between groups and were marked with different letters in the table*

ProDesign R and ProDesign Logic files are identically manufactured, differing only in the cutting direction. PDR is active when rotating counterclockwise and ProDesign Logic is active clockwise. Greater taper can negatively influence the fatigue resistance of the file in curved canals [29]. The tip of ProDesign R, ProDesign Logic and WaveOne Gold primary instruments are the same (0.25 mm). However, the taper of these files are different. ProDesign R and ProDesign Logic have a 6% taper over the first 3 mm from the tip, and the WaveOne Gold primary has a 7% taper. This difference explains the greater rigidity and less fatigue resistance found in the WaveOne Gold primary file. It has previously been reported in other studies [21, 22]. 

Another factor that may have influenced our results was performing the glide path before instrumentation with ProDesign Logic. Previous studies have shown that the glide path is important before NiTi rotary instrumentation [30-32]. An increase in canal diameter before instrumentation with a manual or mechanized file creates a path without anatomical interference and is recommended to reduce the risk of procedural errors [24, 33]. This step can significantly reduce apical transportation, original canal shape deviations and fracture risk of the instrument during preparation, allowing less experienced professionals to achieve the same results as a specialist [29, 34, 35]. In our study, the use of the glide path file in the ProDesign Logic group allowed the instrumentation file to penetrate with less tension and more ease, contributing to better results regarding instrumentation time. Also, it may have been a determining factor in the non-occurrence of fractures of the ProDesign Logic files during instrumentation. Even with evidence demonstrating that reciprocating motion is more resistant to cyclic fatigue [10, 17, 36], we believe that the increase in canal diameter may increase cyclic fatigue resistance of rotating instruments as observed in the results of ProDesign Logic and ProDesign R files.

Different cross-sections may also influence the results since the greater the cross-sectional area corresponds to greater flexural rigidity and torsion [37]. The WaveOne Gold primary file has a cross-sectional design of a decentralized parallelogram with alternating contact at a single point on the wall of the root canal. The ProDesign files have a modified S-shaped cross-section, with contact at two points on the wall. Previous study [38] comparing the cross-sectional area of WaveOne Gold primary and Reciproc R25 (VDW, Munich, Germany) files, shows that the Reciproc file, even with S-cross section, had a larger area than the WaveOne Gold primary. This fact can be attributed to larger taper of Reciproc R25 [38]. The ProDesign files have similar morphological characteristics as the Reciproc files, but with a smaller taper, which may have directly influenced their greater resistance to the WaveOne Gold primary.

The flexibility and resistance properties of the instruments can also be affected by the alloy from which the instrument is manufactured [39]. Thermo-mechanical treatment of NiTi files offers significant benefits regarding the effectiveness and safety of endodontic instruments [5]. Resistance to cyclic fatigue behaves differently depending on the manufacturing process of NiTi alloys. The instrument can have machining defects, created by different surface finishing patterns, which influence its lifespan [14, 40, 41]. The CM-Wire alloy instruments have a crystalline structure mainly in the martensite phase and are manufactured through a unique thermo-mechanical process that allows the control of the material shape memory [5]. This manufacturing process makes the files extremely flexible and resistant to cyclic fatigue [5]. In our study, the instrument alloy type may have been the determining factor in the higher cyclic fatigue resistance of the ProDesign Logic and ProDesign R files compared to the WaveOne Gold primary file. 

## Conclusion

The use of continuous rotational motion in canals with a glide path provided the ProDesign Logic instrument shorter instrumentation time. The cyclic fatigue resistance of ProDesign R and ProDesign Logic instruments was superior to WaveOne Gold. The thermal treatment of the instrument’s alloy, its cross section, and the glide path seems to influence the cyclic fatigue resistance.
